# 

**Published:** 1895-10

**Authors:** 


### TO SUBSCRIBERS—IMPORTANT.

About the time you read this those of you who owe for any part of a year, or more, subscription will receive a bill for the full amount (including the subscription year in which the bill is sent).

We only send these bills once, each October, the time when you should find it most convenient to pay.

If not just ready to pay, please do not forget the bill.

We are very much obliged to those who keep up their payments.

Those who have failed to pay for several years will receive extra pressure soon.

Any honest, legitimate excuse for not paying will be accepted. There is no better journal of its class, but it needs the help of all who owe it to make it worth our time to run it. M. B. H.

---

MISS WINNIE DAVIS, "Daughter of the Confederacy," has written a book entitled "The Veiled Doctor." It is said to be a true story from the life of a physician, embellished, no doubt, by the writer's imagination.

